# Reduced sister chromatid cohesion acts as a tumor penetrance modifier

**DOI:** 10.1371/journal.pgen.1010341

**Published:** 2022-08-22

**Authors:** Jun Wang, Holly R. Thomas, Yu Chen, Stefanie M. Percival, Stephanie C. Waldrep, Ryne C. Ramaker, Robert G. Thompson, Sara J. Cooper, Zechen Chong, John M. Parant

**Affiliations:** 1 Department of Pharmacology and Toxicology, University of Alabama at Birmingham Heersink School of Medicine, Birmingham, Alabama, United States of America; 2 Department of Genetics, University of Alabama at Birmingham Heersink School of Medicine, Birmingham, Alabama, United States of America; 3 Informatics Institute, University of Alabama at Birmingham Heersink School of Medicine, Alabama, United States of America; 4 Hudson Alpha Institute for Biotechnology, Huntsville, Alabama, United States of America; St Jude Children’s Research Hospital, UNITED STATES

## Abstract

Sister chromatid cohesion (SCC) is an important process in chromosome segregation. ESCO2 is essential for establishment of SCC and is often deleted/altered in human cancers. We demonstrate that *esco2* haploinsufficiency results in reduced SCC and accelerates the timing of tumor onset in both zebrafish and mouse p53 heterozygous null models, but not in p53 homozygous mutant or wild-type animals. These data indicate that *esco2* haploinsufficiency accelerates tumor onset in a loss of heterozygosity (LOH) sensitive background. Analysis of The Cancer Genome Atlas (TCGA) confirmed ESCO2 deficient tumors have elevated number of LOH events throughout the genome. Further, we demonstrated heterozygous loss of *sgo1*, important in maintaining SCC, also results in reduced SCC and accelerated tumor formation in a p53 heterozygous background. Surprisingly, while we did observe elevated levels of chromosome missegregation and micronuclei formation in *esco2* heterozygous mutant animals, this chromosomal instability did not contribute to the accelerated tumor onset in a p53 heterozygous background. Interestingly, SCC also plays a role in homologous recombination, and we did observe elevated levels of mitotic recombination derived p53 LOH in tumors from *esco2* haploinsufficient animals; as well as elevated levels of mitotic recombination throughout the genome of human ESCO2 deficient tumors. Together these data suggest that reduced SCC contributes to accelerated tumor penetrance through elevated mitotic recombination.

## Author summary

Tumorigenesis often involves the inactivation of tumor suppressor genes. This often encompasses an inactivation mutation in one allele and loss of the other wild-type allele, referred to as loss of heterozygosity (LOH). The rate at which the cells lose the wild-type allele can influence the timing of tumor onset, and therefore an indicator of a patient’s risk of cancer. Factors that influence this process could be used as a predictive indicator of cancer risk, however these factors are still unclear. We demonstrate that partial impairment of sister chromatid cohesion (SCC), a fundamental component of the chromosome segregation in mitosis and homologous recombination repair, enhanced tumorigenesis. Our data suggest this is through elevated levels of mitotic recombination derived p53 LOH. This study emphasizes the importance of understanding how impaired SCC, mitotic recombination rates, and LOH rates influence cancer risk.

## Introduction

Genomic alterations including missegregation, aneuploidy and micronuclei formation, are hallmarks of cancers and associated with poor patient outcomes [1]. These genomic alterations often occur due to mitotic error; however mutational drivers of these genomic instabilities in tumors are unclear [2–4]. Defects in microtubule attachment and spindle assembly checkpoint have been demonstrated to contribute to genomic instability and cancer predisposition [5,6]. Sister chromatid cohesion (SCC) is another essential process required for equal segregation of chromatids into daughter cells during mitosis and is a probable target for mutations that induce these genomic instabilities. At the core of mitotic SCC, is the cohesin ring, made up of a multiprotein complex that includes SMC1a, SMC3, RAD21, and SA1/2, which lasso the sister chromatids together. ESCO2 establishes SCC around the sister chromatids as they are synthesized during S-phase, whereas Sororin and SGOL1 protect the established SCC until mitosis [7,8]. During metaphase of mitosis the microtubule tension on the kinetochore aligns the sister chromatid into a metaphase plate. During the transition from metaphase to anaphase, the cohesin ring is cleaved by Separase, allowing equal segregation of the sister chromatids to each daughter cell. While SCC is most well-studied with regard to chromosome segregation, it also influences several other biological processes, including DNA repair/homologous recombination [9–13]. The involvement of compromised SCC in cancer has been less well studied since complete loss of SCC triggers cell cycle arrest, apoptosis and/or cellular senescence, which is not conducive to cellular viability, and therefore viewed as disadvantageous to a tumor [14]. Further, mild SCC dysfunction is also suggested to be toxic to the cell due to the need for centromeric cohesion to establish bi-orientation of kinetochores [15]. Hence, SCC is considered an all or none process regarding cellular viability. That said, missense mutations in some components of the SCC have been identified in human tumors, however the mechanistic importance of these mutations remains unknown [16,17].

Li Fraumeni Syndrome (LFS) is an autosomal dominant cancer predisposition syndrome, due to germline heterozygous mutations in p53, in which 50% of patients will succumb to a tumor by age 30[18]. Part of the etiology of tumor formation in LFS, as well as sporadic tumors, is that the first allele has a p53 mutation, and the second undergoes loss of wild-type allele (Loss Of Heterozygosity, LOH) often through whole chromosome deletion/duplication or mitotic recombination [19]. Mouse and zebrafish heterozygous p53 null models have recapitulated this early tumor susceptibility, as well as the LOH of the wild-type allele in tumors [20–24]. Further, murine LFS models suggest that within a cohort of p53 heterozygous animals the timing of tumor onset correlates with the prevalence of p53 LOH in tumors [25]; i.e. the trend is that early-onset tumors often have p53 LOH while late tumors often do not. This suggests that the rate of p53 LOH or prevalence of LOH events influences tumor penetrance. Further, the frequency of tumors with p53 LOH in p53 heterozygous null mice is strain-dependent, in that p53 LOH in a mixed C57/129 strain is ~50%, while in a BALB/c background it is ~96%, suggesting there are undefined genetic factors that can impact the rate of p53 LOH. Note tumor onset was earlier in the BALB/c mice, and the overall higher frequency of mitotic recombination, suggesting difference in DNA repair influence the frequency of p53 LOH and timing of tumor onset [26]. While not explicitly examined with respect to p53 LOH, inactivation of RecQ helicases, such as WRN or BLM, have been shown to increase the rate of mitotic recombination and therefore act as a LOH modifier [27–29].

Our data indicate that reduced SCC, resulting from haploinsufficient loss of *esco2* or *sgo1*, can be tolerated at an organismal level; but leads to accelerated tumor onset in a *p53* heterozygous null LOH sensitive background. Further, the reduced SCC contributes to low-level chromosomal instability in somatic tissues. However, it is elevated mitotic recombination, not chromosomal instability, derived LOH that drives early tumor formation in the *p53* heterozygous null background. TCGA analysis of human cancer corroborates these findings.

## Results

### ESCO2 is frequently deleted/altered in a variety of cancer types

**Fig 1:**
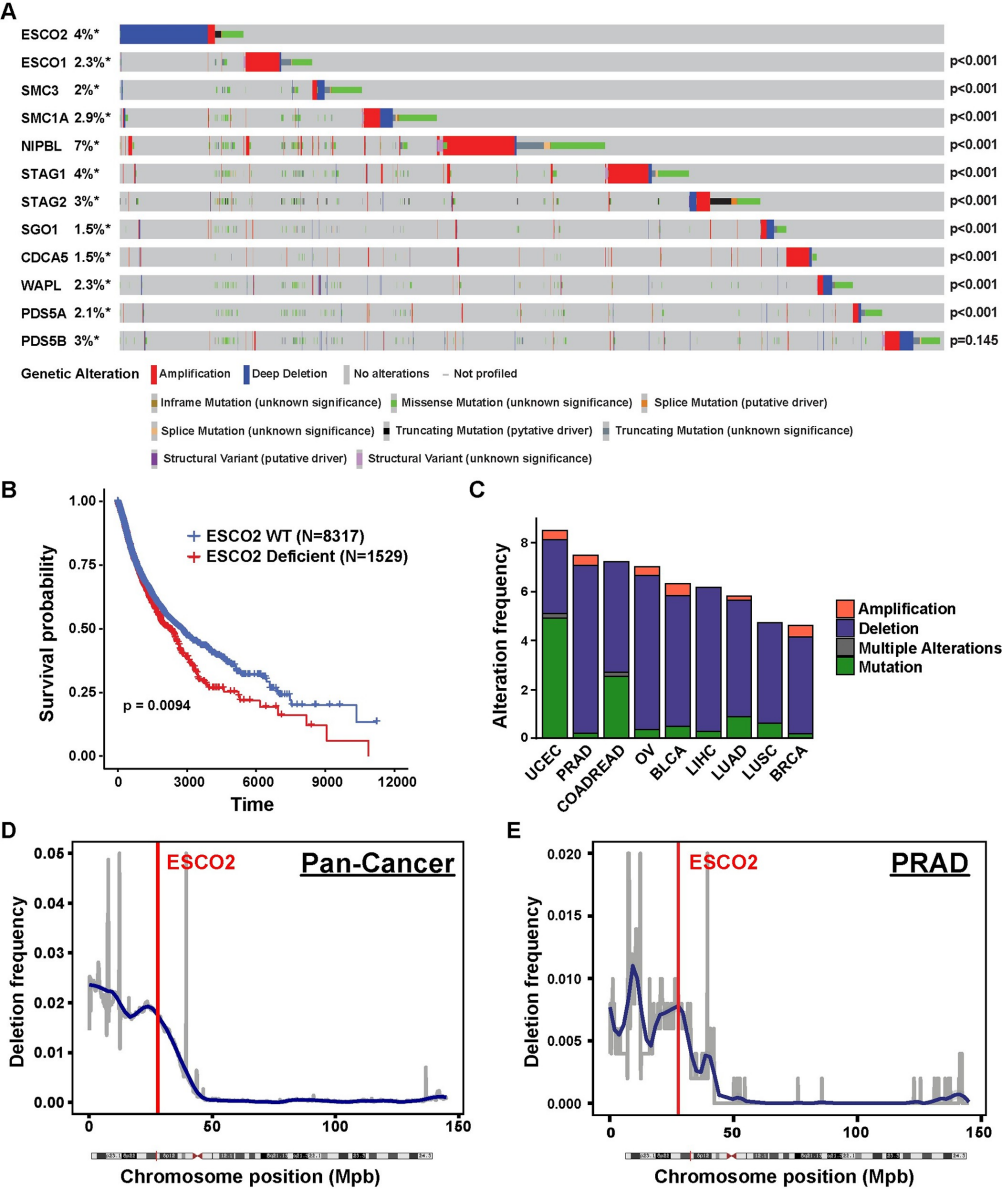
Esco2 deficiencies are common in multiple tumor types and associated with poor patient survival. (A) Oncoprint plot of genetic alterations in ESCO2 and other SCC-associated genes in TCGA dataset. N = 10950. Number of samples with each mutation: N_(ESCO2)_ = 404, N_(ESCO1)_ = 252, N_(SMC3)_ = 216, N_(SMC1A)_ = 322, N_(NIPBL)_ = 743, N_(STAG1)_ = 429, N_(STAG2)_ = 374, N_(SGO1)_ = 165, N_(CDCA5)_ = 159, N_(WAPL)_ = 248, N_(PDS5A)_ = 227 and N_(PDS5B)_ = 375. P-values shown in the figure indicate significance of co-occurrence with ESCO2 from one-sided Fisher Exact test. (B) Kaplan-Meier survival analysis of patients with ESCO2 deficient (N = 1529) versus ESCO2 WT (N = 8317) tumors within TCGA dataset samples. P-value was determined by log-rank test. (C) Stacked bar plot indicating the percentage of patients with an ESCO2 deletion (blue), amplification (red) or mutation (green) and multiple alterations (grey) in uterine corpus endometrioid (UCEC), prostate (PRAD), colorectal (COADREAD), ovarian (OV), bladder (BLCA), liver (LIHC), lung adenocarcinoma (LUAD), lung squamous (LUSC), and breast cancer (BRCA) in the TCGA dataset. Cancer type is ordered based on the genetic alteration frequency. Number of samples with mutation/ total samples in each cancer type: 45/529 UCEC, 37/494 PRAD, 43/594 COADREAD, 41/584 OV, 26/411 BLCA, 23/372 LIHC, 33/566 LUAD, 23/487 LUSC, 50/1084 BRCA. The frequency of deletion in 100 kbp windows throughout Chromosome 8 in 1,111 pan-cancer type (Figure D, N = 11203) and prostate adenocarcinoma (PRAD, Figure E, N = 502) cancer patients. A region is considered as deleted if Log^2^ (Copy Number/2) < -1. Blue line shows smoothed deletion frequency. Red vertical line indicates the locus of ESCO2 gene.

We surveyed The Cancer Genome Atlas (TCGA) database for prevalence of genetic alterations in key sister chromatid cohesion (SCC) factors and found that ESCO2 was most frequently deleted or mutated gene in many cancers **(Fig 1A)**. Further patients with ESCO2 alterations have significantly lower probabilities of survival **(Fig 1B)**. We observed that approximately 5–8% of patients within nine of the most common cancers show alterations in ESCO2 and that these alterations are predominantly deletions and a few protein-altering mutations **(Fig 1C)**. The deletions are defined as at least 50% reduction of gene copy number, suggesting that ESCO2 may be a haploinsufficient tumor suppressor gene. However, ESCO2 resides on chromosome 8p21, and in many human cancers, including liver, breast, prostate, ovarian, uterine, colorectal, bladder and lung cancers, recurrent deletion of genes on the p arm of chromosome 8 is observed **(Fig 1D and 1E)**. This recurring deletion has been associated with advanced tumor progression and poor patient survival [30–32]. This region appears rich in tumor suppressor genes [33]. For example, in breast cancer, genes between 8p21-23 represent five of the top ten most deleted genes [34]. Within this region, there are established tumor suppressors (DLC1, DOK2 and LZTS1), as well as potential tumor suppressor genes (CSMD1, MTUS1, and MSR1), and many other genes not established in cancers [35–40]. Some of these genes, including ESCO2, may be passengers, deleted purely based on their proximity to tumor suppressors, while others may represent novel genes with a role in cancer formation and progression. ESCO2 is important for the establishment of SCC following new synthesis of the sister strand during S-phase of the cell cycle [7]. ESCO2 has not previously been implicated in human cancers, but its function certainly suggests it could be important, and it appears to be at one of the sub-peaks of this deletion region **(Figs 1D, 1E and S1)**. In further support of ESCO2 playing a role in tumorigenesis, some Roberts Syndrome patients (RBS) who carry autosomal recessive germline inactivating mutations in ESCO2, display early-onset cancer predisposition [41–43].

### ESCO2 deficiency results in accelerated tumor onset in LOH-sensitive animal models and elevated LOH in human tumors

**Fig 2:**
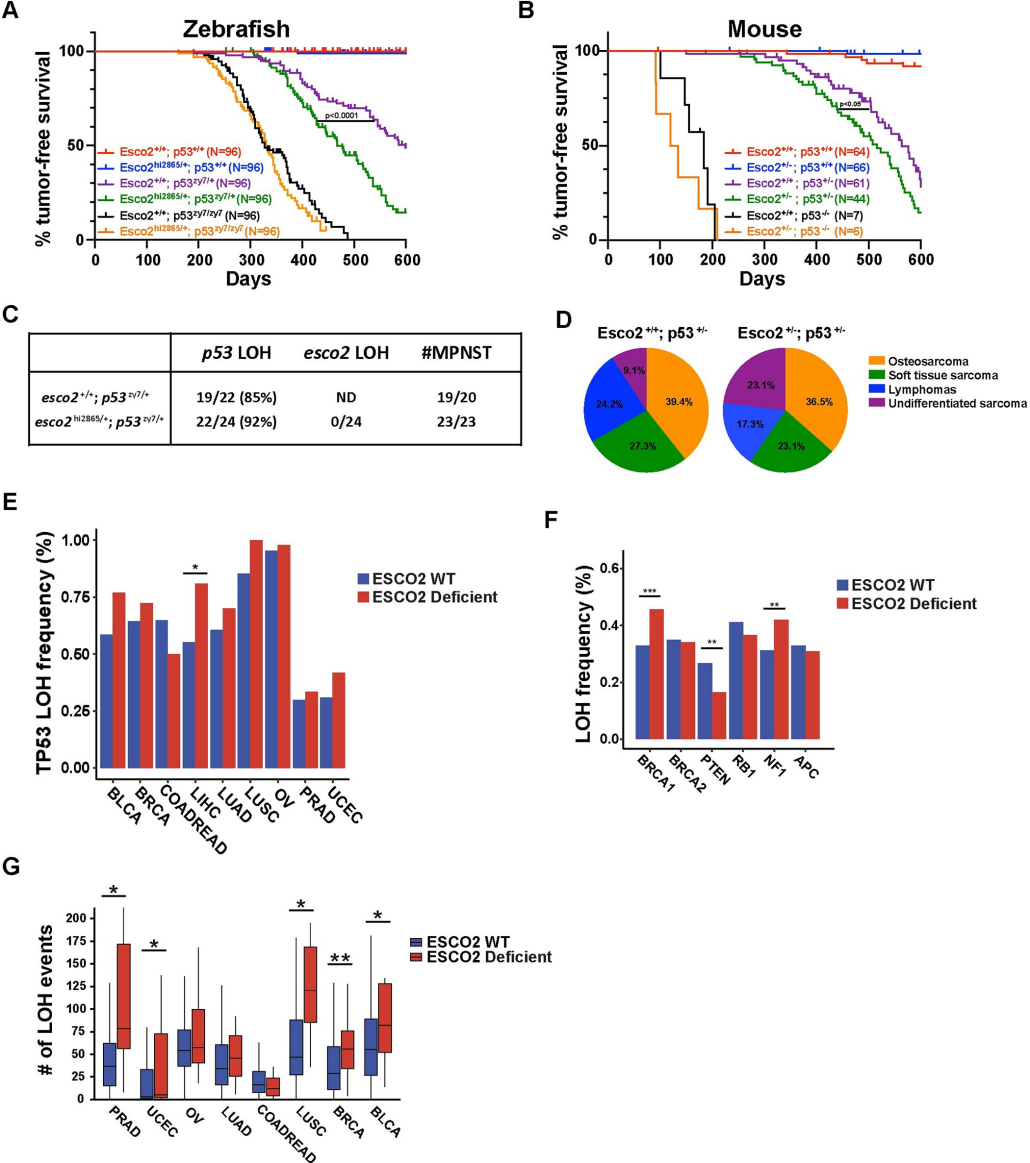
Esco2 deficiencies accelerate tumor onset in a LOH-sensitive background. (A) Zebrafish Kaplan-Meier curves for tumor-free survival for wild-type, *esco2*^*2865/+*^, *p53*^*J19/+*^, *esco2*^*2865/+*^*; p53*^*J19/+*^, *p53*^*J19/J19*^, and *esco2*^*2865/+*^*; p53*^*J19/J19*^ cohorts. Compared cohorts were established by natural single pair breeding of *esco2*^m/+^ x AB (wild-type strain), *esco2*^m/+^; *p53*^m/m^ x AB, or *esco2*^m/+^; *p53*^m/m^ x *p53*^m/m^ parents (all cohorts were n = 96). The p-value = <0.0001 when comparing *p53*^*J19/+*^ with *esco2*^*2865/+*^*; p53*^*J19/+*^ cohorts based on Log-rank (Mantel-Cox) test. (B) Mouse Kaplan-Meier curves for tumor-free survival for wild-type, Esco2 ^+/-^, p53 ^+/-^, Esco2 ^+/-^; p53 ^+/-^ and p53 ^-/-^ cohorts (cohorts with p53^+/+^ and p53^+/-^ background were n>60 and cohorts with p53^-/-^ background were n>6). The p-value = <0.05 when comparing *p53*^*+/-*^ with *esco2*^*+/-*^*; p53*^*+/-*^ curves based on Log-rank (Mantel-Cox) test. (C) Frequency of *p5*3 wild-type loss of heterozygosity (LOH) and *esco2* wild-type LOH in zebrafish tumors; as well as the frequency of tumors being Malignant Peripheral Nerve sheath tumors (MPNST). (D) Pie charts showing tumor spectrum in Esco2^+/+^; p53^+/-^ (left panel) and Esco2^+/-^; p53^+/-^ (right panel) mice. No statistically significant in tumor spectrum with Chi-square test. (E) Percentage of patients with LOH on TP53 in TCGA cancer samples with or w/o ESCO2 mutation/deletion. The number of WT and deficient samples in each tumor were indicated. BLCA: 393 WT + 13 Deficient; BRCA: 935 WT + 29 Deficient; COADREAD: 482 WT + 38 Deficient; LIHC: 333 WT + 21 Deficient; LUND: 495 WT + 10 Deficient; LUSC: 478 WT + 8 Deficient; OV: 383 WT + 46 Deficient; PRAD: 481 WT + 6 Deficient; UCEC: 479 WT + 43 Deficient. * indicates p<0.05 from Fisher’s exact test. (F) Percentage of patients with LOH covering in tumor suppressor gene PTEN, BRCA1, BRCA2, RB1, NF1 and APC in the TCGA dataset. N = 4,126 for ESCO2-WT cohort and N = 193 for ESCO2-deficiency cohort. ** and *** indicate p<0.01 and p<0.001 with Fisher’s Exact test. (G) The number of LOH events per tumor sample in TCGA cancer samples with or w/o ESCO2 mutation/deletion. The number of WT and deficient samples in each tumor were indicated: PRAD: 481 WT + 6 Deficient; UCEC: 479 WT + 43 Deficient; OV: 383 WT + 46 Deficient; LUAD: 495 WT + 10 Deficient; COADREAD: 482 WT + 38 Deficient; LUSC: 478 WT + 8 Deficient; BRCA: 935 WT + 29 Deficient; BLCA: 393 WT + 13 Deficient. * and ** indicate p<0.05 and p<0.01 from Mann-Whitney test.

To decipher if ESCO2 loss is a driver of tumorigenesis, we wanted to determine if ESCO2-deficient animals are tumor predisposed. Both mouse and zebrafish homozygous ESCO2 null animals are embryonic lethal[44, 45], therefore to determine if reduction in ESCO2 specifically contributes to tumorigenesis, we generated and monitored tumor formation in cohorts (N = 96/cohort) of *esco2*^+/+^ and *esco2*^hi2865/+^ (hi2865 allele is an intron 1 gene trap that results in >95% knockdown of transcript) zebrafish in a tumor sensitized *p53* mutant background, *p53*^zy7/+^ (zy7 allele is a thymine to cytosine transition in codon 164 in the p53 DNA binding domain that results in the inability of DNA binding)[22, 44]. Within all zebrafish tumor cohort studies to reduce the influence of background genetic variability, zebrafish cohorts to be compared, were generated from a single pair of zebrafish. Therefore, these two cohorts were generated from an *esco2*^hi2865/+^; *p53*^zy7/zy7^ crossed to a wild-type AB strain zebrafish. We observed statistically significant acceleration of tumor onset **(Fig 2A)** in the *esco2* heterozygous mutant cohort (T_50_ = 467 days in *esco2*^hi2865/+^; *p53*^zy7/+^ cohort vs. 561 days in the *p53*^zy7/+^cohort; p<0.0001 based on log-rank test) suggesting loss of *esco2* is a tumor driving event. However, these experiments were performed in a tumor-sensitized background and did not indicate if *esco2* haploinsufficiency acts as an autonomous tumor suppressor gene. Therefore, we also monitored cohorts of *esco2*^+/+^ and *esco2*^hi2865/+^ zebrafish in a p53 wild-type background, derived from a single pair of *esco2*^hi2865/+^ crossed to wild-type AB. However, over a 2-year period only 2 of 96 *esco2*^hi2865/+^ animals developed a tumor compared to 0 of 96 wild-type animals **(Fig 2A)**, suggesting that if *esco2* does act as an autonomous tumor suppressor gene it has low penetrance. These data indicate that haploinsufficiency for *esco2* enhances tumorigenesis only in a p53 heterozygous background. Loss of the wild-type *p53* allele (loss of heterozygosity (LOH); p53^m/+^ ➔ p53^m^ or ^m/m^) is an important step during tumorigenesis in *p53* heterozygous mutant humans (Li Fraumeni Syndrome) and animals (zebrafish and mouse p53 knockouts) [20,22,46–51]; therefore we postulated that *esco2* heterozygosity may enhance tumor formation in a *p53* heterozygous animal by accelerating *p53* LOH. If true, *esco2* heterozygosity would not enhance tumor formation in a *p53* homozygous background (insensitive to *p53* LOH). Therefore, we monitored a cohort of *p53*^zy7/zy7^ and *esco2*^hi2865/+^; *p53*^zy7/zy7^ zebrafish and found a non-significant difference in tumor enhancement in the *p53 p53*^zy7/zy7^; *esco2*^hi2865/+^ cohort compared to *p53*^zy7/zy7^ cohorts created from single pair of *esco2*^hi2865/+^; *p53*^zy7/zy7^ x *p53*^zy7/zy7^ (**Fig 2A**; T_50_ = 330 days in the *esco2*^hi2865/+^; *p53*^zy7/zy7^ cohort vs. 320 days in the *p53*^zy7/zy7^ cohort; p = 0.526 based on Log-rank test). This finding suggests that *esco2* heterozygosity influences tumor onset only in a LOH sensitive (p53 heterozygous) background. To determine if these observations were also true in mice, we generated similar cohorts of mice using a p53 null allele and a Esco2 null allele (deletion of exon 4) [49,52]. The mouse cohort data is consistent with the zebrafish data, in that Esco2 haploinsufficient loss accelerates tumor onset only in a LOH sensitive background **(Fig 2B**; T_50_ = 516 days in the Esco2^+/-^; p53^+/-^ cohort vs. 563 days in the p53^+/-^ cohort; p = 0.0377 based on Log-rank test). To determine if a higher proportion of the *esco2* deficient derived zebrafish tumors have p53 LOH, we genotyped paired normal and tumor genomic DNA for the p53 allele. The percentage of tumors in the *p53*^zy7/+^ cohort having LOH of the wild-type *p53* allele was 85%, while tumors from the *p53*^zy7/+^; *esco2*^hi2865/+^ cohort was ~92% **(Fig 2C)**. While this is trending, it is a non-significant change in the frequency of LOH-containing tumors. However, measuring LOH in a tumor which is the end-product of tumorigenesis may not reflect the process or rate of LOH in somatic precancerous tissues in these animals. We also determined that the *esco2* gene did not undergo LOH in any of these tumors, suggesting the phenotype is a consequence of a gene dose haploinsufficient state **(Fig 2C)**. Further, we did not observe a change in tumor spectrum, in that 19 of 20 *p53*^zy7/+^ tumors were malignant peripheral nerve sheath tumors (MPNSTs), and 23 of 23 *p53*^zy7/+^; *esco2*^hi2865/+^ tumors were also MPNSTs **(Fig 2C)**. We also evaluated if there was a change in the physical location of the MPNSTs (S2 Fig), but we did not observe a statistical difference between the populations. We did observe more undifferentiated sarcomas (Fig 2D) in the Esco2^+/-^; p53^+/-^ mice cohort, however the timing of this tumor type was not statistically different suggesting this is not the driving force for the accelerated tumor onset. Evaluation of human tumors in the TCGA dataset, indicates that there is statistically significant increase in p53 LOH in Liver hepatocellular carcinoma (LIHC), while there is a trend for increased p53 LOH in ESCO2 deficient tumors compared to ESCO2 proficient tumors **(Fig 2E)**. Further, analysis of other tumor suppressor genes in TCGA data indicated that there are statistically significant increases in LOH of BRCA1, PTEN and NF1 genes in ESCO2 deficient tumors. **(Fig 2F)**. To determine if the increased LOH frequency was only associated with tumor suppressor gene or genes under a selective pressure for LOH, we surveyed the entire genome for LOH frequencies in ESCO2 wild-type and deficient tumors (S1 and S2 Tables). In multiple tumor types we observed significant elevated LOH rates throughout the genome **(Fig 2G)**. Together this indicates that haploinsufficiency in ESCO2 results in a globally higher LOH frequency in tumors.

### Reduced sister chromatid cohesion drives early tumor onset

**Fig 3:**
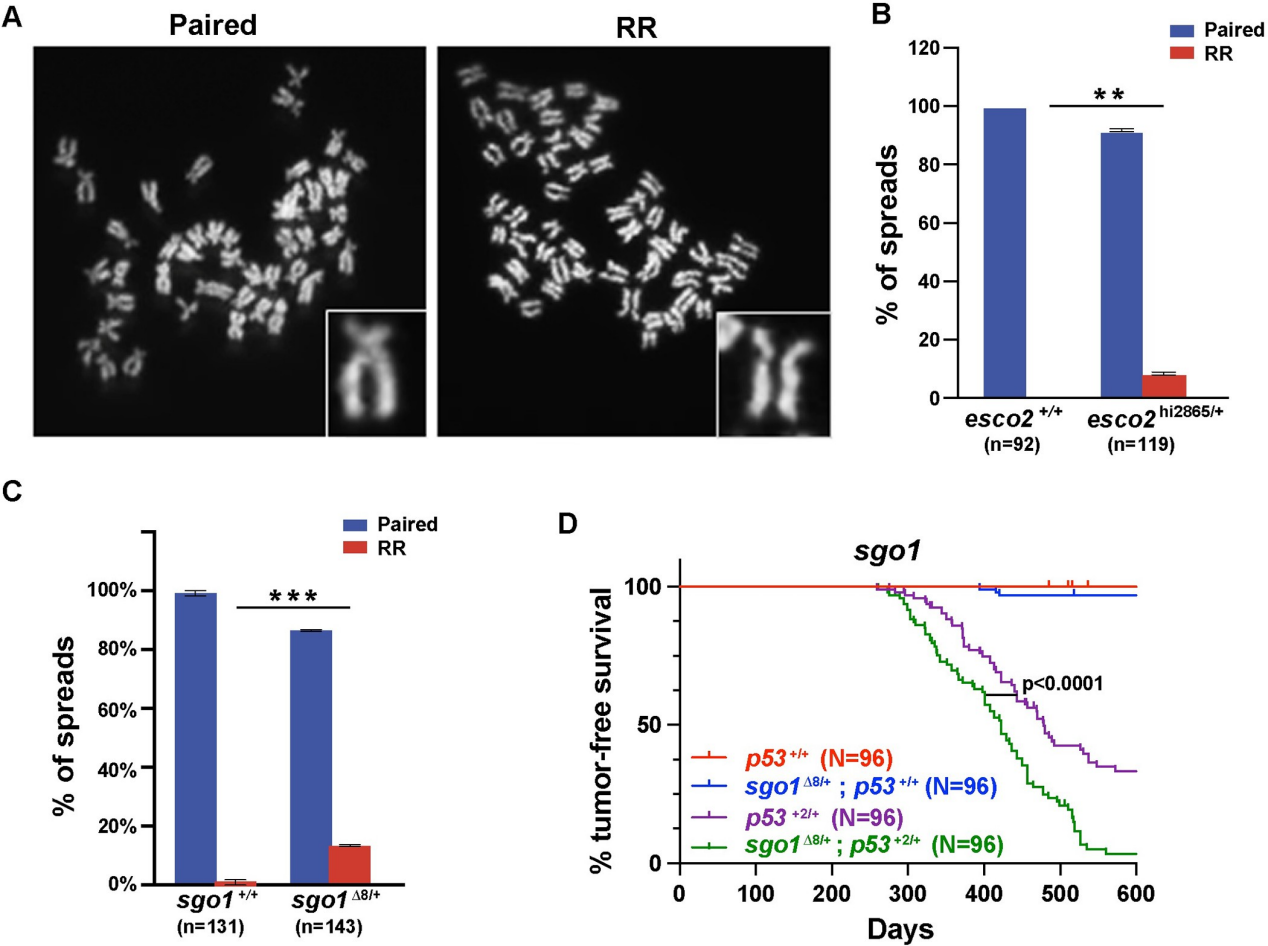
Reduced SCC in esco2 and sgol1 haploinsufficient animals correlates with accelerated tumor onset. (A) Representative images of “paired” and “railroad” (RR) metaphase spreads. Inset shows zoomed-in view of each phenotype. (B) The percentage of metaphase spreads in *esco2*^+/+^ and *esco2*^2865/+^ showing paired or RR phenotypes. Two pools of embryos were used for *esco2*^+/+^ and *esco2*^2865/+^ spreads from two independent experiments. A total of 92 *esco2*^+/+^ and 119 *esco2*^2865/+^ spreads were tallied. (C) The percentage of spreads in *sgo1*^+/+^ and *sgo1*^+/-^ showing paired or RR phenotypes. Three pools of embryos were used for *sgo1*^+/+^ and *sgo1*^m/+^ spreads. A total of 131 *sgo1*^+/+^ and 142 *sgo1*^+/-^ spreads were tallied. ** p-value < 0.01 and *** p-value < 0.001 by paired t-test. (D) Kaplan-Meier curves for tumor-free survival for *p53*^*+/+*^, *sgo1*^*+/-*^*; p53*^*+/+*^, *p53*^*+/-*^ and *sgo1*^*+/-*^*; p53*^*+/-*^ cohorts. Cohorts were established by natural single-pair breeding of *sgo1*^+/-^ X *p53*^+2/+^ parents (all cohorts were n = 96). P-value = <0.0001 when comparing *p53*^*+/-*^ with *sgo1*^*+/-*^*; p53*^*+/-*^ curves based on Log-rank (Mantel-Cox) test.

The most striking observation was that amongst the zebrafish *esco2* heterozygous metaphase spreads, 8.3% displayed a”railroad” (RR) phenotype not observed in the wild-type sibling spreads **(Fig 3A and 3B)**. While similar phenotypes have been described in cell culture, it has never been observed in a live organism [16]. It has been presumed that this phenotype would result in embryonic lethality in a live organism due to the inability to properly orient the kinetochore required for microtubule attachment [15]. However, in this case potentially the viability is associated with the low proportion of cells that have RR. These observations indicate that there is a gene dose-dependent reduction in cohesion establishment in *esco2* heterozygous animals. To address if accelerated tumor onset is specific to *esco2* or reduced SCC, we also generated a zebrafish *sgo1* null allele using CRISPR/Cas9 technique. SGO1 plays an important role in maintaining SCC after establishment by ESCO2. Homozygous null *sgo1* are lethal **(S3 Fig)**, however *sgo1* heterozygous mutant animals are viable and display low-level RR (13.4%) in metaphase spreads similar to *esco2* heterozygous mutants **(Fig 3C)**. This led us to ask would the reduced SCC in *sgo1* mutants also predisposes to early tumor onset in a *p53* heterozygous background. Consistent with the *esco2* haploinsufficient results, heterozygous loss of *sgo1* resulted in statistically significant accelerated tumor onset in a *p53* heterozygous background (**Fig 3D**, T_50_ = 422 days in the *sgo1* ^Δ8/+^; *p53*^+2/+^ cohort vs. 478 days in the *p53*^+2/+^ cohort; p <0.0001 based on Log-rank test). Together this provides two independent genetic models of reduced SCC that are associated with accelerated tumors in a LOH sensitive background.

### Low-frequency chromosome segregation defect in *esco2* haploinsufficient animals

**Fig 4:**
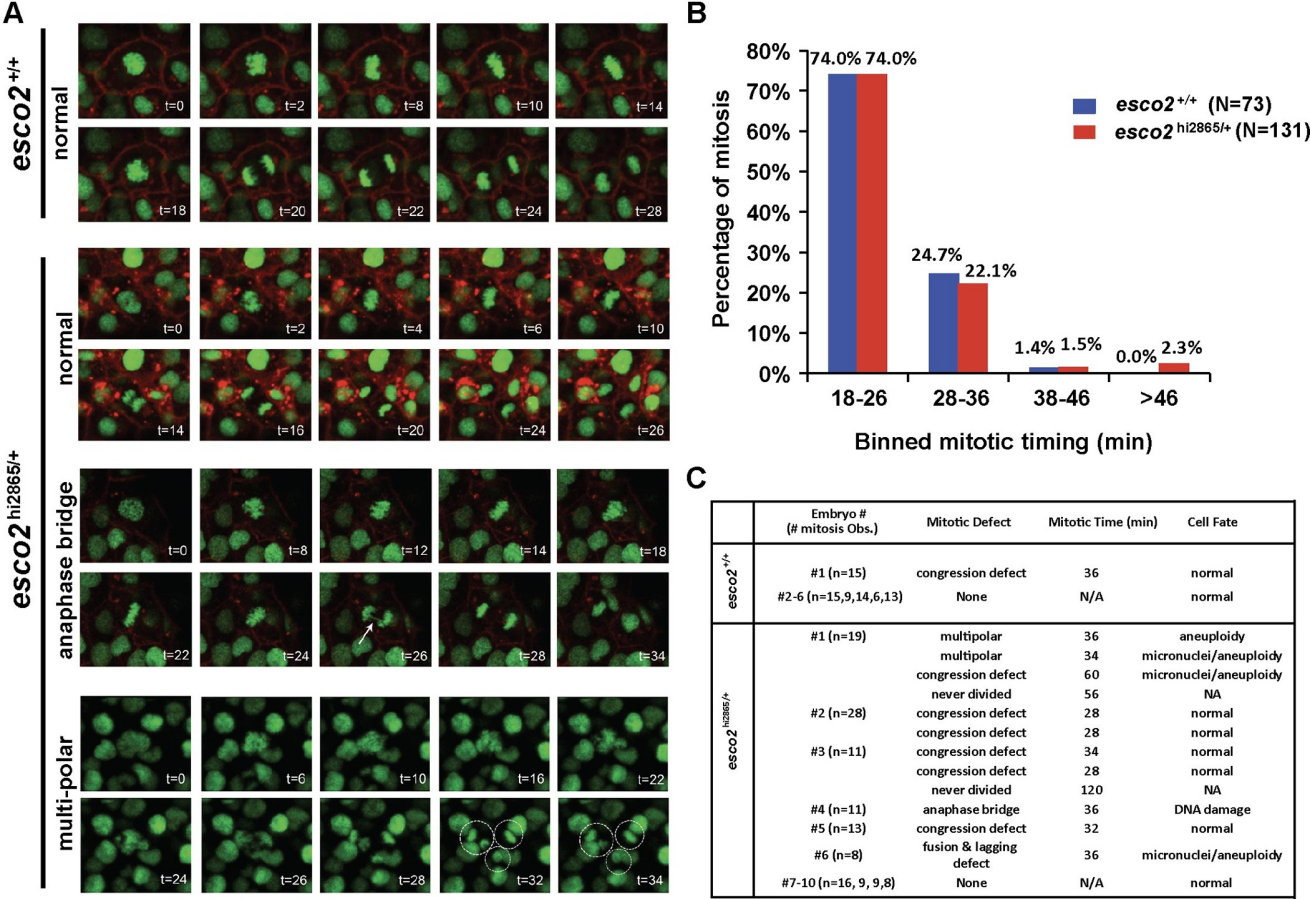
Elevated mitotic segregation errors in esco2 haploinsufficient embryos. (A) in-vivo confocal imaging of H2A.F/Z-EGFP and CaaX-mCherry mRNA injected embryos at 24 hours post-fertilization (hpf) for two hours. Representative images of normal and defective mitoses in *esco2*^+/+^ and *esco2*^m/+^. Arrow in anaphase bridge time-lapse points towards the anaphase bridge formed. Dotted circular in tri-polar time-lapse represents the three future nuclei that will occur. CaaX-mCherry was removed in tri-polar time-lapse for better visualization. t = time in minutes. (B) Division time calculated for each division in six *esco2*^+/+^ and ten *esco2*^m/+^embryos using two-hour imaging time-lapse data from each embryo. The percentage of cells was calculated for each bin category. N = 73 for *esco2*^+/+^ and N = 132 for *esco2*^m/+^. (C) Table representing mitotic defects and the associated mitotic timing and cell fate observed in six *esco2*^+/+^ and ten *esco2*^m/+^embryos.

Chromosome missegregation is one mechanism by which *p53* LOH has been described to occur in tumors [53–56], and we previously observed high levels of chromosome missegregation in the *esco2*^hi2865/hi2865^ animals [44]. To determine if chromosome missegregation occurs in *esco2*^hi2865/+^ animals, we monitored 73 mitoses in six wild-type sibling embryos and 132 mitoses in ten *esco2* heterozygous embryos using a single-cell in-vivo imaging procedure [57]. The majority of mitoses in wild-type embryos were error-free (72 of 73; **Fig 4A**) and were of normal duration (74% were 18–26 minutes; **Fig 4B**). We did observe one abnormal mitotic event that resulted in a congression defect, slightly lengthened mitotic duration, but no observable segregation errors **(Fig 4C and S1 Video)**. We also observed that some wild-type mitoses were mildly longer in length (~25% were 28–36 minutes in length; **Fig 4B**). In *esco2* heterozygous mutant embryos, while the majority of divisions appear normal (120 of 132; **Fig 4A**), we observed 12 mitoses with errors, five (~4%) of which had clear chromosome missegregation events, and two that never exited mitosis within our observation window. These events are summarized in **Fig 4C** and include one anaphase bridge **(Fig 4A and S2 Video)**, six congression defects **(S3 Video)**, two multipolar divisions **(Fig 4A and S4 Video)**, two prolonged delays in metaphase with no division observed (>50min, and >120 min; **S5 Video**), and one cell fusion leading to multiple lagging defects **(S6 Video)**. These events would suggest mild defects in microtubule attachment (lagging chromosomes and congression defects) and/or centrosome duplication (multi-polar divisions). Further, in 3 of 132 (2.3%) mitoses, we observed a severe mitotic delay (60 min, >50 minutes, and >120 minutes; **Fig 4B and 4C**) indicative of mitotic defects resulting in a prolonged spindle assembly checkpoint. Together these data indicate that as early as embryogenesis, haploinsufficient loss of *esco2* contributes to chromosome instability, which could be the driving force for more p53 LOH events.

### Somatic micronuclei frequencies during embryogenesis do not correlate with timing of tumor onset

**Fig 5:**
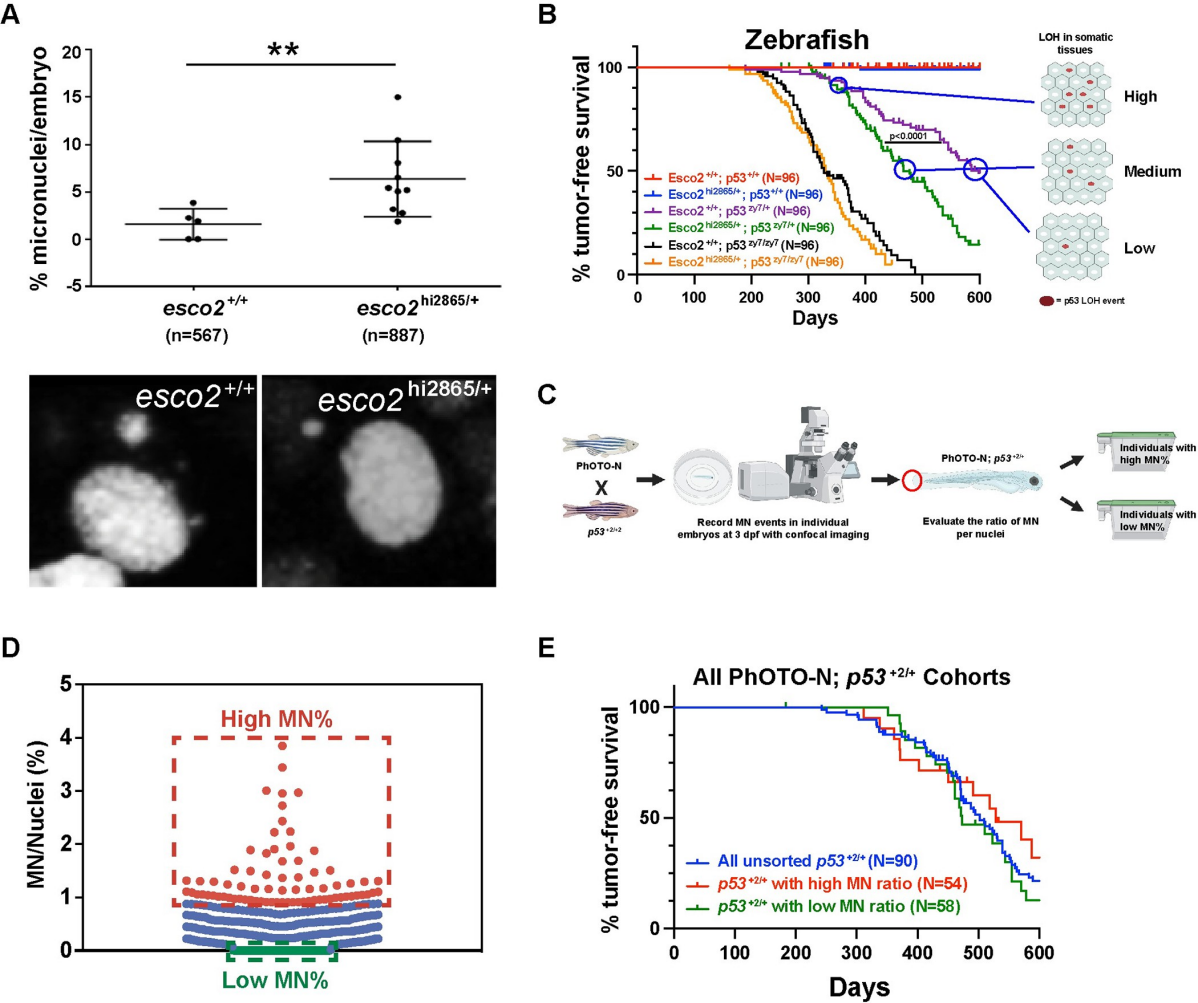
Elevated micronuclei during embryogenesis does not contribute to enhanced tumor onset. (A) Micronuclei (MN) during interphase were counted in six *esco2*^+/+^ and ten *esco2*^2865/+^ H2A.F/Z-EGFP mRNA injected embryos at 24hpf using two-hour live imaging time-lapse data from each embryo. Each dot represents an embryo measured. Percentage MN/embryo was calculated based on the total number of micronuclei per total nuclei present in the time lapse at t = 0. Total nuclei are indicated in parenthesis. Mean ± SD, ** p-value < 0.01 based on unpaired t-test. Representative black and white images of micronuclei in each genotype are shown below. (B) Proposed model where the proportion of cells with MN would correlate with p53 LOH and timing of tumor formation. (C) Experimental workflow to establish high and low MN cohorts, using confocal living-imaging of 3-dpf *p53*^+2/+^; TG^PhOTO-N/+^ embryos. Figure B and C were created with BioRender.com. (D) Dot-blot of Micronuclei frequency in individual 3-dpf *p53*^+2/+^; TG^PhOTO-N/+^ embryos. Individuals deemed part of the high MN cohort were highlighted with red (MN%>0.9%) and individuals in the low MN cohort were highlighted with green (MN% = 0). (E) Zebrafish Kaplan-Meier curves for tumor-free survival for *p53*^+2/+^; TG^PhOTO-N/+^ unsorted (n = 90), *p53*^*+2/+*^; TG^PhOTO-N/+^ with high NM ratio(n = 54) and *p53*^*+2/+*^; TG^PhOTO-N/+^ with high NM ratio (n = 58). P-value is not significance when comparing each other based on Log-rank (Mantel-Cox) test.

Micronuclei (MN) are often observed in cancers, and are derived from ungrouped chromosomes, often lagging chromosomes, during telophase when the nuclear envelope is reformed around chromatid material. MN are an indicator of a mitotic chromosome segregation defect, resulting in one cell gaining chromosomes while the sister cell losing chromosomes which could contribute to p53 LOH. Consistent with our chromosome segregation analysis, we observed a significantly higher number of micronuclei in 1 dpf *esco2* heterozygous mutant embryos (Avg. ~6%) versus the wild-type embryos (Avg. ~2%; **Fig 5A**). Interestingly, there was strong variability in the percentage of cells with micronuclei within embryos of the same genotype, ranging from 0–3.9% in wild-type embryos and 1.9%-15% in *esco2* heterozygous-mutant embryos **(Fig 5A)**. This is also consistent with there being variations in the number of segregation errors observed between embryos of the same genotype (**Fig 4C**; up to 4 missegregation within a scanned field in embryo No.1 verse no events in 4 embryos (No.7-10)). This MN variability and higher levels of MN in *esco2* heterozygous animals spurred a hypothesis that animals with higher proportion of cells with MN, would have more frequent p53 LOH events in precancerous somatic cells, therefore a larger pool of cells that could become tumor-forming, which would translate into earlier tumor onset **(Fig 5B)**. To test this hypothesis, we confocal imaged live 3-dpf p53 heterozygous embryos for the MN frequency using a PhOTO-N chromatin labeling transgenic line **(Fig 5C)**. The TG^PhOTO-N^ transgenic lines ubiquitously expressed H2A-dendra fluorescent protein, which allows for imaging chromosome dynamics as well as determining MN frequency. We used the 3-dpf timepoint to improve viability of embryos following the imaging procedure. We imaged 446 embryos and selected the top 15% MN frequency (0.91% to 3.85% frequency) as the high MN cohort and the bottom 15% (no MN observed) as the low MN cohort **(Fig 5D)**. These cohorts as well as an un-imaged p53 heterozygous cohort (with TG^PhOTO-N^ background) were monitored for tumor formation. Surprisingly we did not observe a significant difference in the high vs low MN cohort **(Fig 5E)**. This suggests that chromosome missegregation and elevated MN in embryos are not the driving force behind elevated LOH and earlier tumor onset in *esco2* haploinsufficient animals. Note these experiments do not address the impact of chromosome missegregation and elevated MN that occurs in adult somatic tissues.

### Elevated mitotic recombination derived LOH

**Fig 6:**
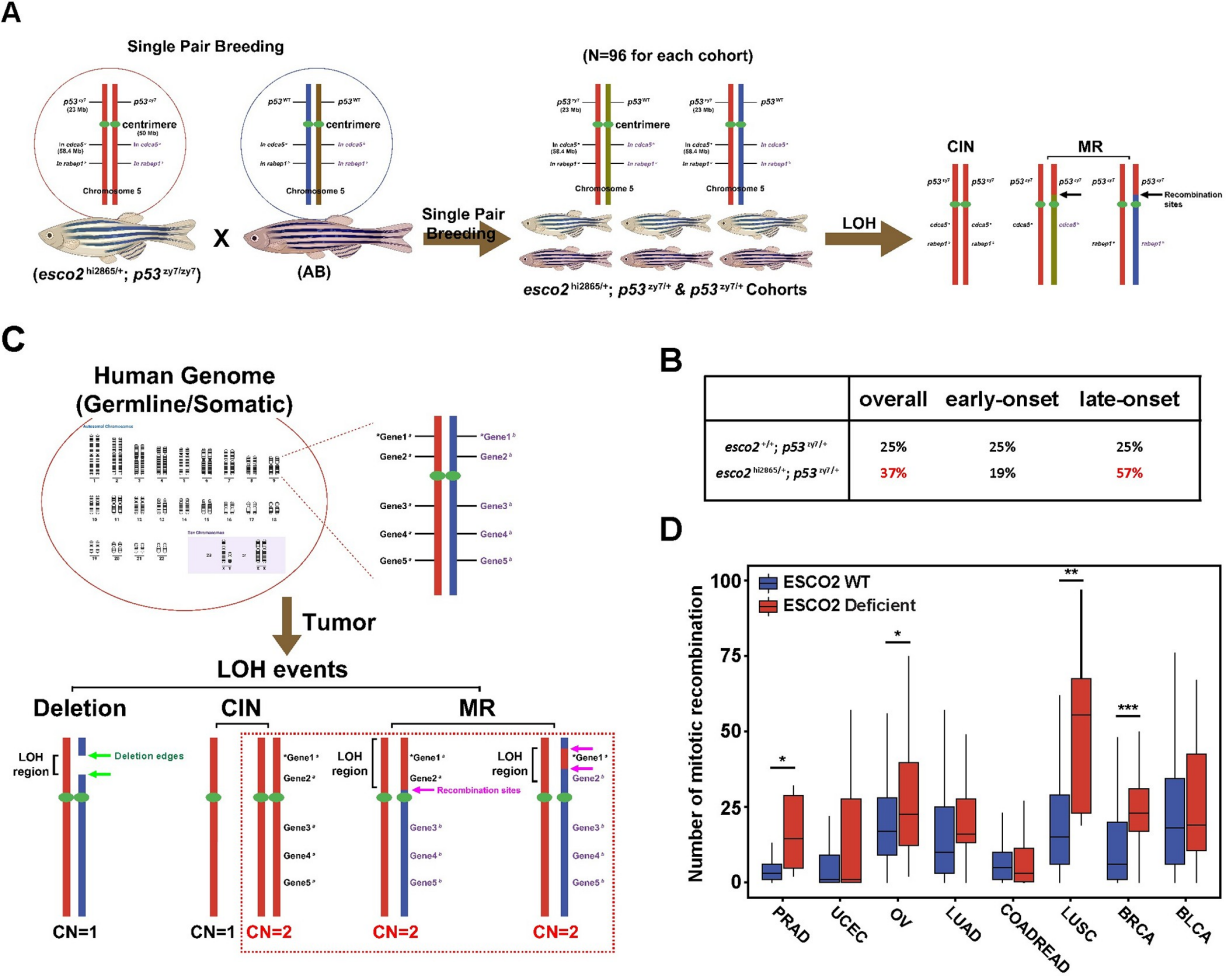
Reduced SCC allows for elevated mitotic recombination derived LOH. (A) Schematic of determining LOH mechanisms/types in *p53* heterozygous tumors. The haplotype of nearby p53 marker in tumor and normal paired samples, cooccurring with p53 wild-type or mutant gene are labeled (left). (B) A table showing the percentage of mitotic recombination (MR) in *esco2*^+/+^; *p53*^zy7/+^ and *esco2*^hi2865/+^; *p53*^zy7/+^ tumors. (C) Schematic of determining the mitotic recombination with TCGA database. Figure A and C were created with BioRender.com. (D) The number of mitotic recombination in TCGA cancer samples with or w/o ESCO2 mutation/deletion. Genome-wide data was downloaded from NCI Genomic Data Commons. The number of WT and deficient samples in each tumor were indicated: PRAD: 481 WT + 6 Deficient; UCEC: 479 WT + 43 Deficient; OV: 383 WT + 46 Deficient; LUAD: 495 WT + 10 Deficient; COADREAD: 482 WT + 38 Deficient; LUSC: 478 WT + 8 Deficient; BRCA: 935 WT + 29 Deficient and BLCA: 393 WT + 13 Deficient. *, ** and *** indicate p<0.05, p<0.01 and p<0.001 from Mann-Whitney test.

p53 LOH can also be derived from mitotic recombination (MR) in which the region containing p53 becomes homozygous while the region between the MR site and the centromere as well as the opposite arm remains heterozygous **(Fig 6A)**. Since our high and low MN cohort experiment suggested the whole chromosome instability (CIN) is not the driving force behind p53 LOH, we wanted to investigate mitotic recombination as a mechanism of p53 LOH in our *esco2* heterozygous zebrafish tumors. For this we identified a polymorphic SNP in *rabep1* or *cdca5* in normal tissues, that resided on the opposite arm of p53 containing chromosome **(Fig 6A)**. Using these markers, we observed an increased rate (37%vs25%; **Fig 6B**) of heterozygosity maintained at *rabep1* or *cdca5* in tumors derived from *esco2* heterozygous animals, indicative of increased MR. Since we observed the largest difference in the survival curves in the population of animals forming tumors later, we decided to analyze the MR rate of tumors that occur in the first half (<T_50_) and the second half (>T_50_) of the tumor cohorts. Interestingly, while in the *esco2* wild-type cohort the rate of MR was 25% in the first and second half, we observed a much higher MR rate (57%) in the second half of the *esco2* heterozygous derived tumor cohort. These data suggest MR-derived LOH is a driving force for the overall earlier tumor onset in the *esco2* heterozygous cohort (T_50_ = 467 days in esco2^hi2865/+^; p53^zy7/+^ cohort vs. 561 days in the p53^zy7/+^cohort). To determine if human cancers that have ESCO2 deficiencies also have higher MR rates, we analyzed TCGA data to define all LOH loci that maintained a 2N copy number **(Fig 6C)**, this would exclude regional deletions or whole chromosome deletion, as well as amplifications. We then plotted these across the chromosome to define MR events. From this analysis we determined that there were more MR events, in multiple cancer types, in ESCO2 deficient tumors compared to proficient tumors **(Fig 6D)**. Together these data suggest that reduced SCC enhances the rate of MR which drives LOH-sensitive tumorigenesis.

## Discussion

These data indicate that gene dose-dependent loss of *esco2* or *sgo1* results in reduction in SCC and enhanced tumor predisposition. Interestingly, amongst viable (majority die in utero or postnatally) Roberts Syndrome (RBS) patients, which are homozygous null of ESCO2, they also display reduced SCC and early onset tumor occurrence [41–43]; supporting the fact that reduced SCC is tumor-promoting across species. Further, our analysis of the TCGA data strongly supports that ESCO2 loss in tumors is associated with elevated mitotic recombination and elevated tumor suppressor inactivation. While this study has focused on ESCO2, SCC is a complex network of pro-cohesion factors and anti-cohesion factors (yeast genetics suggests over 350 genes involved) [58], in which the synergistic or antagonistic effects of combined mutations/polymorphisms in these genes can create varying degrees of SCC dysfunction amongst a population of individuals and potentially define variability of tumor penetrance between individuals. Consistent with this, alterations in other SCC factors such as Separase overexpression, STAG2 loss, SGO1 haploinsufficiency, and others have been associated with tumorigenesis [16,59–61]. Additionally, deficiencies in the tumor suppressor retinoblastoma (RB1) have recently been tied to SCC loss and suggestive of a mechanism by which RB loss promotes tumorigenesis [62]. SCC involvement in tumorigenesis is also interesting in that there is a yin-yang relationship between the cohesion establishment and antiestablishment processes. In yeast, deletion of Eco1, the homolog of ESCO2, is lethal; while concomitant loss of Eco1 and WAPAL, or PDS5, circumvent this lethality [63–66]. This suggests that there could be a window where ESCO2 or another establishment molecule is lost, which would encourage MR and selection, and then a second window where an anti-establishment factor is lost to restore stability of an aneuploid genome. However, this Yin-Yang relationship is yet to be demonstrated during tumorigenesis. Together these data emphasize that defects in SCC can strongly influence tumorigenesis.

While chromosome instability has been the focus of studies looking at the consequence of defects in SCC, some studies have pointed to increased rates of homolog recombination versus sister chromatid recombination [67,68]. In most normal circumstances, due to the cohesin rings, the sister chromatids are in proximity and therefore the preferred source for homologous recombination and produces a “perfect” repair. However, in situations of reduced SCC, chromosome homologs now have better access to each other, and homologous recombination can occur between homolog chromosomes [10,69]. This can result in multiple repair outcomes, including a crossover event and/or gene conversion events. Following mitosis this recombination between homologs can result in chromosome regions becoming homozygous (mitotic recombination (MR)), resulting in a LOH event. This is consistent with our tumor data in which reduced SCC is associated with elevated MR events. Changes in MR rates are most well demonstrated in recombinase deficient individuals and animal models, such as Bloom syndrome proteins (BLMs) or Werner syndrome helicase (WRN), but we demonstrated that this may also be true for SCC reduced models and syndromes.

Interestingly the variability in the age of tumor onset within LFS is great, with some patients developing tumors within the first year of life, while others have been described to be tumor-free at the age of 74. The timing of tumor onset is influenced by many different factors, including environment and genetic heterogeneity. While these are clearly influential, isogenic animal models under controlled environmental conditions can exclude these factors and focus on genetic aspects. In a *p53*-centric view, the difference between the survival curves of a *p53*^zy7/zy7^ and *p53*^zy7/+^ cohort (at T_50,_ a difference of 241 days vs. 561 days respectively) is rationalized as the time required for the 2^nd^ *p53* hit to occur (*p53* LOH; *p53*^zy7/+^ ➔ *p53*^zy7 or zy7/zy7^). The difference between *p53*^zy7/+^ and *p53*^+/+^ curves is the time required for the first *p53* hits to occur (*p53*^+/+^ ➔ *p53*^zy7/+^). However, the reason that the *p53*^zy7/zy7^ tumors are not observed immediately at time 0 is due to the time needed for the additional required tumor-promoting mutations to occur (for example Ras and/or other). We demonstrate that reduced SCC through haploinsufficient loss of *esco2* or *sgo1*, decreased the time between *p53*^zy7/zy7^ and p53^zy7/+^ curves (at T_50_, a 147-day difference) suggesting that reduced SCC enhances the rate of *p53* LOH events. Further, since there was no change in survival curves in the *p53*^zy7/zy7^ background, *esco2* haploinsufficiency is not an added oncogenic hit. It is also interesting that in these isogenic and environmentally controlled models that there is a vast difference in the time of tumor onset within these cohorts. For mouse trp53 heterozygous null in a C57BL6/J background, the first tumors are observed at ~150 days, while the later tumors are at >600 days [48]. What influences this difference? We would speculate that the proportion of cells in an organism with stochastically derived p53 LOH in somatic tissues influences the timing of tumor formation. While measuring p53 LOH in tumors is relatively easy due to the clonal source of the genomic DNA, the ability to measure p53 LOH in somatic cells has been close to impossible. However recent advances in single-cell haplotyping might provide a first-time view of p53 LOH rates in somatic tissue and variability between tissues and individuals. If true, individual evaluations of p53, or other tumor suppressor genes, LOH events in somatic tissues of a patient could provide a cancer risk for those individuals.

It is worth noting that some aspects of tumorigenesis with regards to p53 status are different in mouse and zebrafish. In zebrafish, the day distance at the T_50_ between the *p53*^zy7/zy7^ and *p53*^zy7/+^ is shorter suggesting they are more prone to LOH of the wild-type allele than mouse. Interestingly, the time for p53^-/-^ mice to form tumors is much earlier than *p53*^zy7/zy7^ zebrafish and would suggest that these “hits” in mouse are acquired more readily or require less number of hits than in zebrafish. Further, the impact of reduced SCC-derived p53 LOH may have different impact on tumorigenesis between mice and zebrafish. In zebrafish the *p53*^zy7/+^; *esco2*^hi2865/+^ and *p53*^zy7/+^; *sgo1*^Δ8/+^ curve progressively diverges from the *p53*^zy7/+^ curve with time, while in mouse they run parallel. Since the accelerated curves in mouse and zebrafish only occur in the p53 heterozygous animal, inactivation of the wild-type p53 allele is important in the accelerated tumorigenesis. However, potentially the impact that inactivation of p53 has on tumorigenesis is different between species; i.e. inactivation of p53 in zebrafish may continually progresses acquisition of “hits”, while in mouse they are already acquired. Alternatively, increased genomic LOH with reduced SCC maybe adds the additional “hits”, independent of p53 status, such that accelerated tumorigenesis occurs, while the necessary “hits” are already acquired in the mouse. Together this may suggest that the zebrafish model is more strongly influenced by reduced SCC and/or LOH, compared to the mouse model.

## Materials and methods

### Ethics statement

All zebrafish work was performed in the Zebrafish Research Facility (ZRF) of the University of Alabama at Birmingham (UAB). Adult fish and embryos are maintained as described by Westerfield et al (1995) by the ZRF Animal Resources Program which maintains full American Association for Accreditation of Laboratory Animal Care (AAALAC) accreditation and is assured with the Office of Laboratory Animal Welfare (OLAW). All mouse studies were conducted in compliance with the National Institutes of Health Guide for the Care and Use of Laboratory Animals. All animal studies have UAB Institutional Animal Care and Use Committee (IACUC) approval (protocol #20705)

### Mouse lines and maintenance

The Esco2 knockout line was acquired from The European Conditional Mouse Mutagenesis Program (Esco2^tm1a(EUCOMM)Wtsi^ EPD0409_6_A03) [52]. Esco2 conditional allele was generated by crossing the Esco2 Knock out first line to a Rosa26 flp line (Jackson lab #009086). The Esco2 null allele was generated by crossing the conditional allele to a Rosa-CreER line (National Cancer Institute Mouse Repository Strain #01XAB), and injecting Tamoxifen into the pregnant mom to remove exon 4. All Esco2 alleles were maintained on a C57BL6/J background. The p53 KO allele was obtained from Jackson Labs (Strain #002101). The p53 allele was maintained on a C57BL6/J genetic background.

### Zebrafish lines and maintenance

approval. The *esco2*^hi2865^ retroviral insertion allele was obtained from Nancy Hopkins and Jacqueline A. Lees (Massachusetts Institute of Technology, Cambridge, MA) and maintained on the AB wild-type background [70]. The *p53*^zy7^ allele was obtained from Dr. Yost at the University of Utah [22] (a thymine to cytosine transition in codon 164), also maintained on an AB background. TG^PhOTO-N^ (pMTB:memb-Cerulean-2A-H2B-Dendra2, a transgenic line ubiquitously expressing membrane-targeted blue fluorescent protein cerulean and chromatin targeted photoconvertible fluorescent protein Dendra2)[71] transgenic fish was obtained from Periklis Pantazis (ETH, Laboratory of Nano Bio Imaging, through Heidi Hehnly-Chang at SUNY upstate as an intermediary). The p53^+2^ allele [23] was generated and maintained on an AB background. The *sgo1*^Δ*8*^ knock-out allele was generated (see below) and maintained on an AB background.

### Generation of new zebrafish knockout alleles

Gene Knockouts were generated as described previously [72]. gRNA target sites were identified using the Zhang lab gRNA design tool (gRNA sequencing and target sites listed in **S3 Fig**). The CRISPR gRNA sequences were cloned into pDR274 (Addgene NO. 42250). The Cas9 mRNA was transcribed from pT3TS-nCas9n (Addgene No. 46757) [73]. After cloning specific target plasmids/guides into pCS2 variant vector, mRNA was generated by in vitro transcription of NotI-HF linearized DNA using the Invitrogen mMESSAGE mMACHINE SP6 Transcription Kit (Fisher Scientific AM1340) and purified with the MEGAclear Transcription Clean Up Kit (Fisher Scientific AM1908). 1-2nl of sgRNA/Cas9 mRNA was microinjected into the yolk of one-cell-stage wild-type zebrafish embryos. For indel efficiency evaluation, genomic DNA was extracted from ~24 3dpf injected embryos and evaluated with HRM (see below). The remaining embryos (F0s) from the same clutches were raised. Out-of-frame indels identified in F1 progeny with Sanger sequencing were maintained and propagated. To “clean up” genetic background all lines were bred at least 2 generations to the wild-type strain AB.

### Identification of zebrafish mutated alleles

To determine the mutated allele, a small piece of tail was cut from a single F2 heterozygous progeny (of each allele) to extract genomic DNA through incubation at 98°C for 20 min in 40μl 25mM NaOH in a 96-well plate, then neutralized with 40μl of 40mM Tris-HCl. The PCR amplicons were amplified using Takara Ex Taq DNA Polymerase (Takara Bio, RR001A), purified with the Promega Wizard SV Gel and PCR Cleanup System (Promega, A9282), and examined on a 1% agarose gel (for examining alternative splicing) and sequenced by the UAB Heflin Center for Genomic Sciences Sanger Sequencing Core.

### Establishing zebrafish and mouse tumor cohorts

The six paired zebrafish tumor cohorts (*esco2*^+/+^ vs *esco2*^hi2865/+^, *esco2*^+/+^; *p53*^zy7/+^ vs *esco2*^hi2865/+^; *p53*^zy7/+^, and *esco2*^+/+^; *p53*^zy7/zy7^ vs *esco2*^hi2865/+^; *p53*^zy7/zy7^) were established by natural breeding of *esco2*^hi2865/+^ x AB (wild-type strain), *esco2*^hi2865/+^; *p53*^zy7/zy7^ x AB or *esco2*^hi2865/+^; *p53*^zy7/zy7^ x *p53*^zy7/zy7^ parents respectively. Four zebrafish tumor cohorts (*sgo1*^+/+^, *sgo1*^Δ*8/+*^, *sgo1*^+/+^; *p53*^+2/+^, *sgo1*^Δ*8/+*^; *p53*^+2/+^) were established by natural single-pair breeding of *sgo1*^Δ*8/+*^ X *p53*^+2/+^ parents. Each paired cohort consisted of 96 fish and was derived from a single set of parents (a single male and female). At 3 months of age, each fish was genotyped for and then separated into 6 tanks of 16 fish each (for *esco2* cohorts) and 4 tanks of ~24 fish each (for *sgo1* and *p53*^+2/+^ micronuclei cohorts). For the micronuclei (MN) study, three zebrafish tumor cohorts (*p53*^+2/+^ unimaged control, *p53*^+2/+^ with high MN ratio and *p53*^+2/+^ with low MN ratio) were established by natural breeding of *p53*^+2/+2^ x TG^PhOTO-N^ transgenic parents multiple times. *P53*^+2/+^ cohort consisted of 90 fish, *p53*^+2/+^ with high NM ratio consisted of 54 fish and *p53*^+2/+^ with low NM ratio consisted of 58. Six mouse tumor cohorts (Esco2^+/+^, Esco2^+/-^, Esco2^+/+^; p53^+/-^, Esco2^+/-^; p53^+/-^, Esco2^+/+^; p53^-/-^, and Esco2^+/-^; p53^-/-^) were established by natural breeding of Esco2^+/-^; p53^+/-^ X 129 mice (first 4 cohorts) and Esco2^+/-^; p53^+/-^ X Esco2^+/+^; p53^+/-^ mice (last 2 cohorts). Kaplan-Meier analysis was performed using GraphPad Prism software.

### Zebrafish and mouse genotyping

To genotype, tail clippings from each fish were placed in 100 μl ELB (10 mM Tris pH 8.3, 50 mM KCl, 0.3% Tween 20, 0.3% NP40, 1 mg/ml Proteinase K) in 96-well plates. Tail clips were incubated at 55°C overnight to generate genomic DNA, and the plates were then incubated at 95°C for 10 min to inactivate the Proteinase K. PCR reactions contained 1ul of LC Green Plus Melting Dye (BioFire Defense), 1ul of enzyme buffer, 0.2 ul of dNTP Mixture (10mM each), 0.3 ul of MgCl_2_, 0.3 ul of each primer (10uM), 1 ul of gDNA, 0.05 ul of Genscript Taq, and water up to 10ul. For *esco2* allele, PCR amplicons were generated using a universal forward primer: 5′-TTTCACTGTTTCTGCAGGTTG-3′, reverse primer 5′-TAAGGTCTTCGAAGTCTTAACG-3′ to amplify the wild-type, and reverse primer 5′ GGGGGGGGGCCTACAGGTGGGGTCTTTC-3′ to amplify the retroviral insertion allele. For *p53* mutant allele (J19), PCR amplicons were generated using forward primer: 5’-GCGCCTGCTGGTCA-3’, reverse primer 5’-CTGATTGCCCTCCACTCTT-3’. For *p53* +2 allele, wild-type and mutant amplicons were generated using forward primer: 5’-AGTACTTGCCGGGATCGTTT-3’, reverse primer 5’-GTCTCCGGAACAGTGGATGT-3’. For *sgo1* Δ8 allele, wild-type and mutant amplicons were generated using forward primer: 5’-AGCGTTCAGGCCAACAATAA-3’, reverse primer 5’-GCGGGTCTCTCTCTCAGTGT-3’. The PCR reaction protocol was 98°C for 30 sec, then 40 cycles of 98°C for 10 sec, 59°C for 20 sec, and 72° C for 15 sec, followed by 72°C for 1 minute (for *esco2*^hi2865^ allele) and 95°C for 20 sec (for *sgo1*^Δ*8*^, *p53*^zy7^ and *p53*^+2^ allele) and then rapid cooling to 4°C. Following PCR, melting curves were generated and analyzed using the LightScanner instrument (Idaho Technology) over a 65–95°C range. For identifying mouse p53 allele, PCR amplicons were generated using a universal reverse primer for both alleles: 5′-CCCATGCAGGAGCTATTACACA-3′, forward primer to amplify the wild-type allele: 5′-GGTCACCTGTAGTGAGGTAGGG-3′, and forward primer to amplify the mutant allele: 5′-CCTCTGTTCCACATACACTTCA-3′, following the Jackson standard protocol for p53 KO stain #002101.

### Loss of heterozygosity analysis

DNA was extracted from 22 *esco2*^+/+^; *p53*^zy7/+^ and 24 *esco2*^hi2865/+^; *p53*^zy7/+^ zebrafish tumors using the DNeasy Blood & Tissue Kit (Qiagen). LOH analysis was performed for the *esco2* allele using the HRM method described above and for the *p53* allele by sequencing PCR products. Each PCR reaction contained 3 μl Ex Taq Buffer, 2.4 μl dNTPs (2.5mM each), 0.9 ul forward primer (5ˊ-GTGCAGCCCTACACTGGAAT-3′) and reverse primer (5ˊ-GGTCCTACAAAAAGGCTGTGA -3′), 50–100 ng of DNA, 0.15 μl of Ex Taq DNA polymerase and water up to 30ul. PCR conditions were as follows: 9800B0030C for 30 sec, then 40 cycles of 98°C for 10 sec, 56°C for 30 seconds, and 72° C for 30 seconds, followed by 72°C for 4 minutes. Each PCR reaction was analyzed on a 2% agarose gel and purified using the Wizard SV Gel and PCR Clean Up System (Promega). Each sample was sequenced by the UAB Heflin Center for Genomic Science.

### Gross imaging

Zebrafish embryos were dechorionated at described stages with incubation in 0.03% pronase (Sigma P5147) for 5–7 min and anesthetized using 0.4% tricaine. In a 60 x 15 mm Falcon petri dish, embryos are mounted in 1% low melting agarose and gross images were taken on a Nikon SMZ-18 Zoom Stereo Microscope. All images were acquired at the same magnification, exposure time and gain. After each embryo was imaged, embryos were removed from the agarose to generate genomic DNA for genotyping. Further figure processing and analysis were performed using Nikon NIS Element and ImageJ.

### Confocal time-lapse imaging

CaaX-mCherry and H2A.F/Z-EGFP mRNA was transcribed from gift plasmids, pCS2-CaaX-mCherry and pCS2-H2A.F/Z-EGFP from K. Kwan (University of Utah) using mMessage mMachine SP6 kit (Life Technologies). *esco2* heterozygotes were crossed, and zebrafish embryos were microinjected into the yolk of a one-cell-staged embryo with 1 nl of 200 ng/μl CaaX-mCherry and 200 ng/μl H2A.F/Z-eGFP mRNA. At 24 hours post-fertilization (hpf), embryos were screened for fluorescence. Embryos showing a mutant phenotype were excluded. Embryos were manually dechorionated using tweezers and anesthetized using 0.4% tricaine. In a glass-coverslip-bottomed dish, embryos were embedded in a 1% low-melt agarose. Dishes were placed on the Nikon A1 inverted confocal microscope and Z-stack images were taken at designated intervals. Approximately 40-μm Z-stacks (with a 2-μm interval) were obtained every 2 minutes for a total scanning time of 2 hours. After each embryo was imaged, embryos were removed from the agarose to generate genomic DNA for genotyping. All videos were taken using Plan Apo 60x oil 1.4 NA objective. 3D viewing, still shots and videos were assembled and processed using NIS Elements 4.13.00. Division time was calculated by manually counting how many time intervals encompass the division. This number was then multiplied by the time between each Z-stack (2 minutes). Further details are shown in our video manuscript [57].

### Micronuclei counting

Zebrafish embryos were injected with H2A.F/Z-EGFP and CaaX-mCherry mRNA and set up for a time-lapse video. An approximately 40-μM Z-stack was generated with 2-μm steps using a 60x 1.4 NA objective on a Nikon A1 confocal microscope. Using 3D volume rendering in NIS Elements 4.13.00, the frequency of micronuclei in interphase was calculated by dividing the total number of micronuclei observed in the 3D render by the number of nuclei identified in the 3D render. Representative micronuclei images were pulled from the 3D volume rendering of an *esco2*^*+/+*^ and *esco2*^hi2865/+^, CaaX fluorescence was removed, and image was converted to black and white. For the investigation of whether the micronuclei ratio affects the onset of tumor formation, instead of mRNA injection, *p53*^+2/+2^ fish were crossed to TG^PhOTO-N^ transgenic line which ubiquitously express photoconvertible fluorescent protein Dendra2 fused to H2B to detect micronuclei within a cell. The imaging technique and analysis were identical to that using mRNA injections.

### Chromosome spreads

Chromosomes spread protocol was adapted from the Lee group [74]. Approximately 20–30 zebrafish embryos were dechorionated at 24 hpf. Embryos were incubated in 400 ng/ml nocodazole for 2 h in the dark at 28°C. Embryos were then transferred to 1.1% sodium citrate in a 6-cm dish and deyolked. At this point, for genotyping purposes, tails were removed to be genotyped, whereas the remaining embryo heads/trunk were transferred to fresh sodium citrate solution and incubated on ice for 8 min. Next, we performed two washes with a cold 3:1 methanol: acetic acid solution for 20 min each followed by storage at −20°C overnight until genotyping was performed. After fixative procedure, embryos were pooled (10–12 embryos/pool) per genotype and then minced using forceps in a 1:1 methanol: acetic acid solution. Using this mixture, 150 μl of pooled embryos were dropped onto a slide, and 3–5 drops of glacial acetic acid were added. The slide was then exposed to hot vapors (we used boiling water) for about 10 s; then allowed to dry on a hot metal surface (approx. 55°C). After the slide was completely dry, a few drops of Prolong Gold with DAPI (Life Technologies) were added, and the slide was covered with a glass coverslip. Chromosomes were imaged with 100x objective on the Zeiss Axio Imager A2 and analyzed with the Zen 2011 Blue Edition software. Although most spreads were clearly delineated into the ‘paired’ or RR ‘railroad’ categories, if a spread had multiple phenotypes it was categorized by which was most prevalent in that spread. Chromatid number was counted manually from high-resolution images.

### TCGA and human tumor analysis

To obtain an overview of ESCO2 mutational status across cancers, oncoprint plot of ESCO2 and other SCC-associated genes was generated with cBioPortal (https://www.cbioportal.org/) using 10,950 samples from 32 studies under TCGA Pan Cancer Atlas Studies category. The somatic mutation calls, copy number variation calls, and clinical survival data of TCGA samples from all 33 TCGA cancer projects were obtained from TCGA GDC data portal (https://portal.gdc.cancer.gov/). Sample number in each analysis was indicated in figure legend. Patients were classified as ‘ESCO2 Mutant/Deletion’ if there was non-synonymous somatic SNV/indel or copy number loss in the ESCO2 gene region. Survival analysis was performed with R package ‘survival’ and the differences in patient survival status by ESCO2 mutational status were assessed by log-rank test. Genetic alteration frequency of ESCO2 in different cancer types was plotted with cBioPortal using corresponding studies in TCGA PanCancer Atlas Studies. For deletion frequency analysis, the number of copy number loss was counted in each 100kbp bin on the human reference genome and then divided by the total number of patients in the cohort to calculate deletion frequency. Deletion frequencies in each bin were plotted using the R package ‘ggplot2’ and a smoothed line was generated using the Loess function. For genome-wide LOH analysis, LOH calls were downloaded from https://gdc.cancer.gov/about-data/publications/pancan-aneuploidy, which were previously inferred from SNP array and exome sequencing data [75]. The LOH status on BRCA1 and other tumor suppressors was classified by comparing the genomic coordinates of each LOH event and tumor suppressor gene. LOH frequency differences by ESCO2 status (WT vs. Mutant) were assessed by Fisher’s exact test [76]. Number of LOH events was counted for ESCO2 Mutant/Deletion and ESCO2 WT patients in each cancer type and the differences were assessed with Mann-Whitney test. Mitotic recombination frequencies were inferred from the LOH regions with neutral copy numbers excluding chromosome-level events. For CNV burden visualization, CNVs with focal CNV values smaller than -0.5 were categorized as copy number loss.

### Statistical analysis

A combination of GraphPad Prism and R statistical packages was used in generation of all graphs and statistical tests. For the zebrafish work, overall statistical significance was calculated using an unpaired t-test with error bars indicating standard deviation as stated in legend (±). All p-values that were determined to be significant are noted in individual figure legends. Unpaired t-test determined the significantly different values. The Log-rank (Mantel-Cox) test was performed for tumor survival studies. Numbers of embryos and significance values are indicated in the figure legends. For analysis of human TCGA data, the R statistical package was used, and the statistical tests are listed in the relevant methods and figure legends.

## Supporting information

S1 Fig Frequency of deletion in 100kbp windows throughout Chromosome 8 in patients with (A) bladder (BLCA, N = 415), (B) lung squamous (LUSC, N = 503), (C) colorectal (COADREAD, N = 466), (D) ovarian (OV, N = 606), (E) liver (LIHC, N = 378), (F) lung adenocarcinoma (LUAD, N = 532) and (G) uterine cancer (USC, N = 56). Blue line shows smoothed deletion frequency. Red vertical line indicates the position of ESCO2 gene on Chr8.
(TIF)

S2 Fig Pie charts showing the MPNST tumor location in (A) *esco2*^+/+^; *p53*^zy7/+^ and (B) *esco2*^hi2865/+^; *p53*^zy7/+^ (right panel) zebrafish. N number is indicated. There was no statistically significant difference in the tumor location based on Chi-square test.
(TIF)

S3 Fig Generation and validation of a stable *sgo1* mutant in zebrafish. (A) Diagram of the target site in zebrafish *sgo1* genome. gRNA target site in exon 2 of the *sgo1* gene (arrow) and PAM motif (red). We identified multiple alleles and propagated an 8-bp deletion that results in a frame shift at codon 180. (B) genomic DNA sequence chromatogram of *sgo1* heterozygous zebrafish showing the 8-bp deletion in mutant allele. (C) The wild-type and truncated *sgo1* protein. Red indicates the out-of-frame amino acid sequence in mutant allele. (D) Representative gross images of *sgo1*^+/+^ and *sgo1*^-/-^ at 24hpf and 5dpf in a lateral view. 46 out of 75 mutants showing abnormal phenotype at 5 dpf. Scale bar, 1000μM.
(TIF)

S1 Table Genomic coordinates of LOH events in TCGA samples. (XLSX)

S2 Table Number of LOH and MR events in TCGA samples. (XLSX)

S1 Video Time-lapse imaging of mitosis in an esco2+/+ 24 hpf zebrafish embryo in which DNA is labeled green and cell membrane red via H2A.Z/F-EGFP and CAAX-mCherry, respectively. NEB is inferred by the irregular appearance of the nucleus and proceeds towards congression of the sister chromatids to form a metaphase plate. Prior to accurate segregation, a minor congression defect occurs but results in formation of two new daughter cells with no evidence of genomic instability. Video was acquired on a Nikon A1 confocal microscope using a 60x objective. Z-stack of embryo tail was performed through approximately 40μm of tissue, obtaining a z-slice every 3μm. A z-stack was taken every two minutes for two hours. Z-stacks were compiled into a 3D projection where individual divisions were cropped out and put in sequence to show as time-lapse.
(MP4)

S2 Video Time-lapse imaging of mitosis in an esco2+/m 24 hpf zebrafish embryo in which DNA is labeled green and cell membrane red via H2A.Z/F-EGFP and CAAX-mCherry, respectively. NEB is inferred by the irregular appearance of the nucleus and proceeds towards congression of the sister chromatids to form a metaphase plate. Prior to accurate segregation, chromatids are inaccurately attached creating a bridge as the division progresses towards anaphase. The bridge is resolved as cell division is completed. Video was acquired on a Nikon A1 confocal microscope using a 60x objective. Z-stack of embryo tail was performed through approximately 40μm of tissue, obtaining a z-slice every 3μm. A z-stack was taken every two minutes for two hours. Z-stacks were compiled into a 3D projection where individual divisions were cropped out and put in sequence to show as time-lapse.
(MP4)

S3 Video Time-lapse imaging of mitosis in an esco2+/m 24 hpf zebrafish embryo in which DNA is labeled green and cell membrane red via H2A.Z/F-EGFP and CAAX-mCherry, respectively. NEB is inferred by the irregular appearance of the nucleus. As the chromatin proceeds towards formation of a metaphase plate, several congression defects occur. These defects are resolved as the cell progresses through cell division with no evidence of genomic instability. Video was acquired on a Nikon A1 confocal microscope using a 60x objective. Z-stack of embryo tail was performed through approximately 40μm of tissue, obtaining a z-slice every 3μm. A z-stack was taken every two minutes for two hours. Z-stacks were compiled into a 3D projection where individual divisions were cropped out and put in sequence to show as a time-lapse.
(MP4)

S4 Video Time-lapse imaging of mitosis in an esco2+/m 24 hpf zebrafish embryo in which DNA is labeled green and cell membrane red via H2A.Z/F-EGFP and CAAX-mCherry, respectively. NEB is inferred by the irregular appearance of the nucleus. Chromatin forms a “Y” shape instead of a metaphase plate. The cell then divides into three cells instead of two with one of these cells being binucleated. Video was acquired on a Nikon A1 confocal microscope using a 60x objective. Z-stack of embryo tail was performed through approximately 40μm of tissue, obtaining a z-slice every 3μm. A z-stack was taken every two minutes for two hours. Z-stacks were compiled into a 3D projection where individual divisions were cropped out and put in sequence to show as a time-lapse.
(MP4)

S5 Video Time-lapse imaging of mitosis in an esco2+/m 24 hpf zebrafish embryo in which DNA is labeled green and cell membrane red via H2A.Z/F-EGFP and CAAX-mCherry, respectively. NEB is inferred by the irregular appearance of the nucleus. The cell remains in prometaphase for the duration of the two-hour acquisition. Video was acquired on a Nikon A1 confocal microscope using a 60x objective. Z-stack of embryo tail was performed through approximately 40μm of tissue, obtaining a z-slice every 3μm. A z-stack was taken every two minutes for two hours. Z-stacks were compiled into a 3D projection where individual divisions were cropped out and put in sequence to show as a time-lapse.
(MP4)

S6 Video Time-lapse imaging of mitosis in an esco2+/m 24 hpf zebrafish embryo in which DNA is labeled green and cell membrane red via H2A.Z/F-EGFP and CAAX-mCherry, respectively. NEB is inferred by the irregular appearance of the nucleus. Two adjacent cells appear to fuse by transferring a single chromatid between each cell. These cells ultimately divide with evidence of genomic instability. Video was acquired on a Nikon A1 confocal microscope using a 60x objective. Z-stack of embryo tail was performed through approximately 40μm of tissue, obtaining a z-slice every 3μm. A z-stack was taken every two minutes for two hours. Z-stacks were compiled into a 3D projection where individual divisions were cropped out and put in sequence to show as a time-lapse.
(MP4)
