# Supplementary figures and images for: Reduced sister chromatid cohesion acts as a tumor penetrance modifier
Source: PLoS Genet. 2022 Aug 22;18(8):e1010341. doi: 10.1371/journal.pgen.1010341 (PMC9436123; doi:10.1371/journal.pgen.1010341)

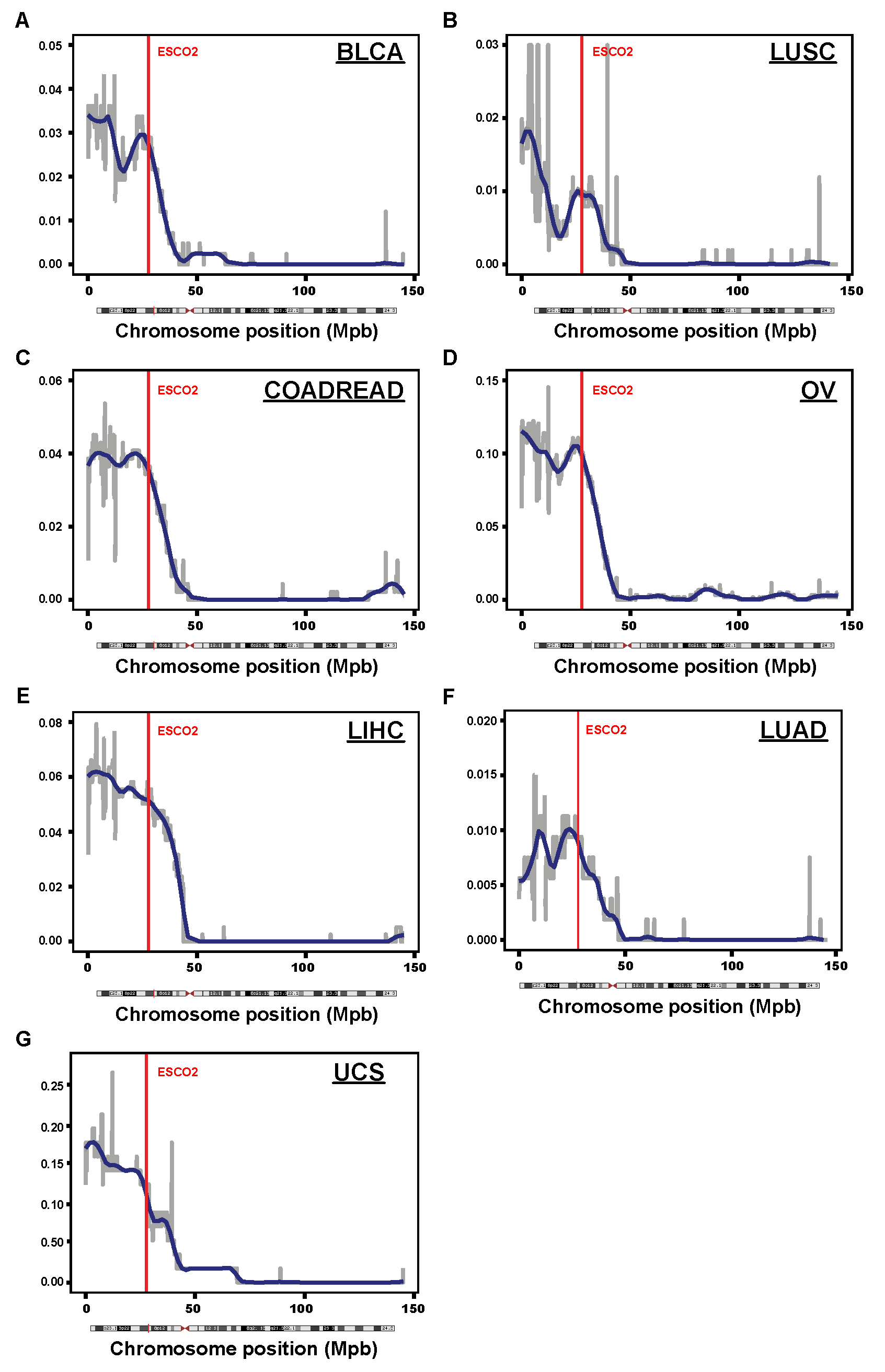

Supplement: S1 Fig — Frequency of deletion in 100kbp windows throughout Chromosome 8 in patients with (A) bladder (BLCA, N = 415), (B) lung squamous (LUSC, N = 503), (C) colorectal (COADREAD, N = 466), (D) ovarian (OV, N = 606), (E) liver (LIHC, N = 378), (F) lung adenocarcinoma (LUAD, N = 532) and (G) uterine cancer (USC, N = 56). Blue line shows smoothed deletion frequency. Red vertical line indicates the position of ESCO2 gene on Chr8. (TIF) [file pgen.1010341.s001.tif]

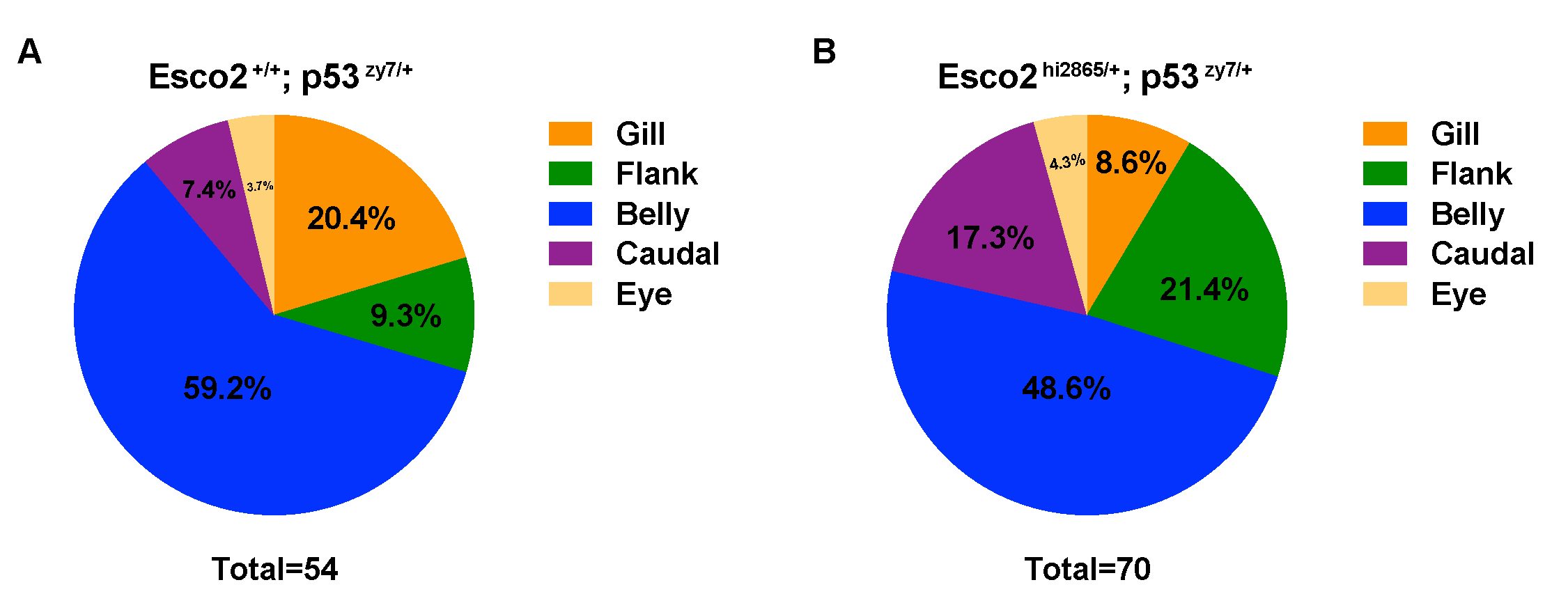

Supplement: S2 Fig — Pie charts showing the MPNST tumor location in (A) esco2+/+; p53zy7/+ and (B) esco2hi2865/+; p53zy7/+ (right panel) zebrafish. N number is indicated. There was no statistically significant difference in the tumor location based on Chi-square test. (TIF) [file pgen.1010341.s002.tif]

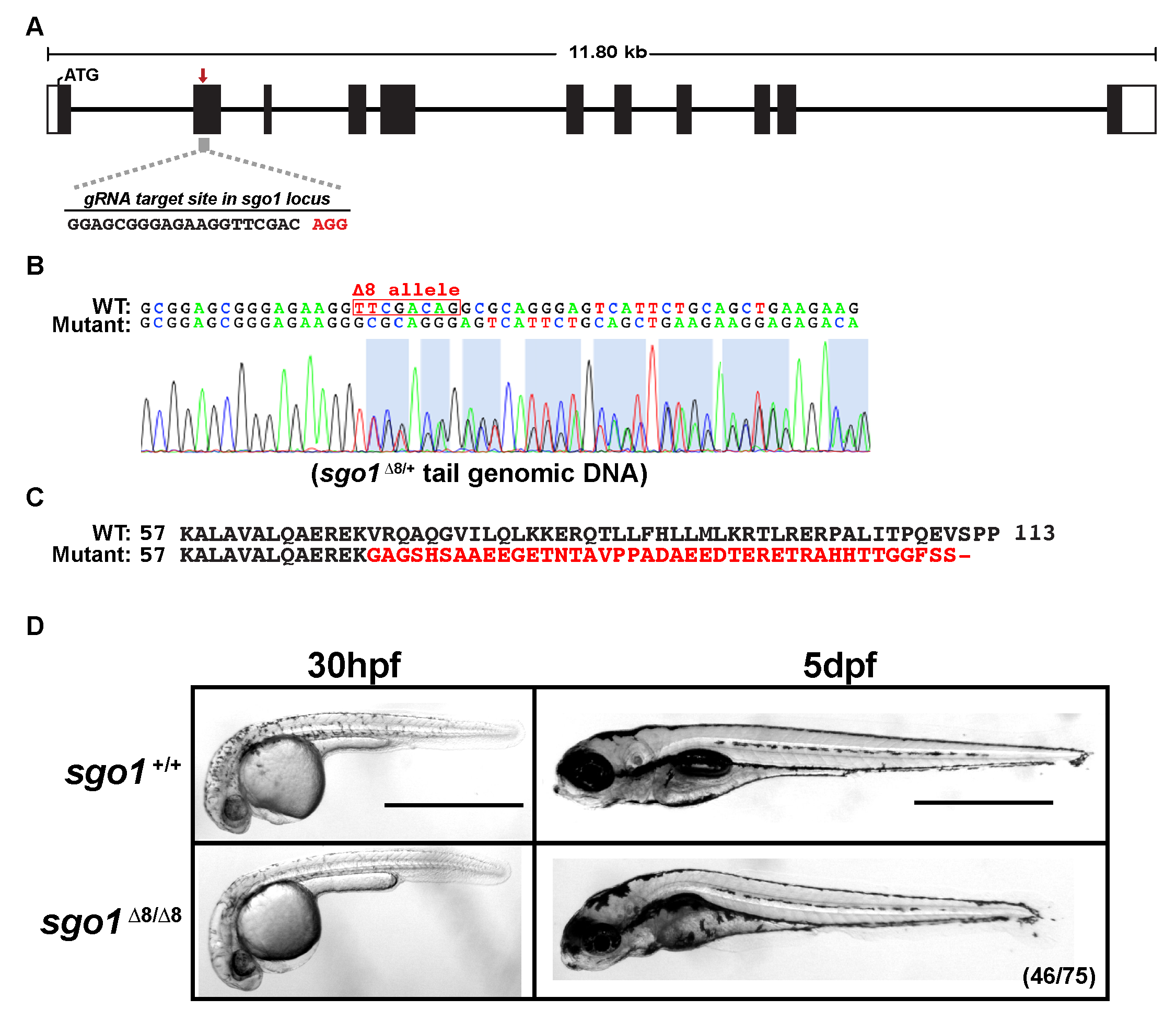

Supplement: S3 Fig — (A) Diagram of the target site in zebrafish sgo1 genome. gRNA target site in exon 2 of the sgo1 gene (arrow) and PAM motif (red). We identified multiple alleles and propagated an 8-bp deletion that results in a frame shift at codon 180. (B) genomic DNA sequence chromatogram of sgo1 heterozygous zebrafish showing the 8-bp deletion in mutant allele. (C) The wild-type and truncated sgo1 protein. Red indicates the out-of-frame amino acid sequence in mutant allele. (D) Representative gross images of sgo1+/+ and sgo1-/- at 24hpf and 5dpf in a lateral view. 46 out of 75 mutants showing abnormal phenotype at 5 dpf. Scale bar, 1000μM. (TIF) [file pgen.1010341.s003.tif]
